# Gorlin Syndrome with Bilateral Polydactyly: A Rare Case Report

**DOI:** 10.5005/jp-journals-10005-1221

**Published:** 2013-10-14

**Authors:** Sonu Acharya, Swagatika Panda, Kanika Singh Dhull, Sujit Ranjan Sahoo, Prayas Ray

**Affiliations:** Reader, Department of Pedodontics and Preventive Dentistry, Institute of Dental Sciences, SOA University, Bhubaneswar, Odisha, India; Senior Lecturer, Department of Oral Pathology and Microbiology Institute of Dental Sciences, SOA University, Bhubaneswar, Odisha India; Reader, Department of Pedodontics and Preventive Dentistry, Kalinga Institute of Dental Sciences, KIIT University, Bhubaneswar, Odisha India, e-mail: kanikasingh.dhull@gmail.com; Senior Lecturer, Department of Oral Pathology and Microbiology Institute of Dental Sciences, SOA University, Bhubaneswar, Odisha India; Assistant Professor, Department of Pedodontics and Preventive Dentistry, SCB Dental College and Hospital, Cuttack, Odisha, India

**Keywords:** Basal cell nevus syndrome, Carcinoma, Odontogenic keratocyst, Gorlin-Goltz syndrome

## Abstract

Gorlin's syndrome is a rare disorder transmitted as an autosomal dominant trait. It is characterized by multiple disorders involving multiple systems. We present a case of 11-year-old male child presenting with multiple odontogenic keratocyst to the dental clinic. Retrograde diagnosis of Gorlin-Goltz syndrome was made after clinical and radiological investigation.

**How to cite this article:** Acharya S, Panda S, Dhull KS, Sahoo SR, Ray P. Gorlin Syndrome with Bilateral Polydactyly: A Rare Case Report. Int J Clin Pediatr Dent 2013;6(3):208-212.

## INTRODUCTION

Gorlin-Goltz syndrome (GGS) also known as nevoid basal cell carcinoma syndrome is a rare autosomal dominant disorder characterized by a wide spectrum of developmental anomalies and neoplasms. ^1^ The incidence of this disorder is estimated to be one in 50,000 to 150,000 in the general population, varying by region.^2^ Males and females are equally affected.^3^

The pathogenesis is attributed to the mutations in the patched tumor suppressor gene (PTCH), mapped to chromosome 9q21-23.^4^ The common manifestations in this syndrome include multiple basal cell carcinomas (BCCs), odontogenic keratocysts, skeletal abnormalities, hyperkeratosis of palms and soles, intracranial ectopic calcifications of the falx cerebri, and facial dysmorphism such as macrocephaly with frontal bossing, cleft lip/palate and severe eye anomalies. Intellectual deficit is present in up to 5% of cases. Various neoplasms like medulloblastomas, meningiomas, ovarian and cardiac fibromas have been also reported.^5,6^ Because of the different systems affected, a multidisciplinary approach of various experts is required for a successful management and usually a dentist makes the initial diagnosis either coincidentally or by virtue of dental complaints. Early diagnosis of the condition and regular follow-up are important for better survival rates from the coexistent diseases. Several case reports^7,8^ and excellent reviews^9,10^ of this condition have been published in the literature. However, the numerous manifestations of the syndrome still appear not to be recognized fully. We are reporting a case of 11-year-old male with the chief complaint of pain and swelling of lower jaw later found to present the other clinical and radiographic findings of Gorlin-Goltz syndrome like pectus excavatum, polydactyly, bifid rib, Falx cerebri calcification and precocious cataract.

## CASE REPORT

**Fig. 1:**
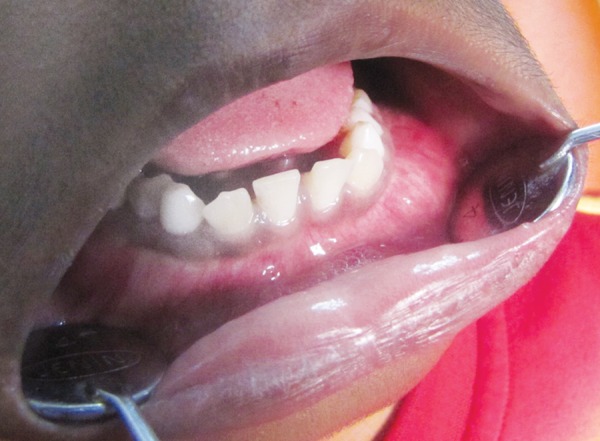
Intraoral view showing buccal swelling in mandible

**Fig. 2:**
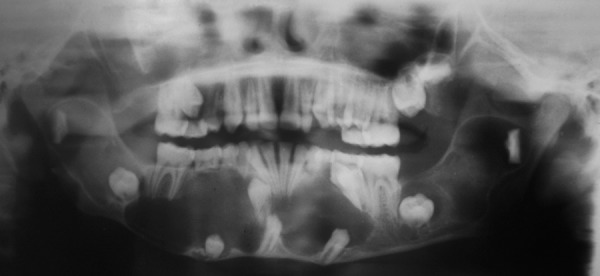
Panoramic radiograph

An 11-year-old child presented with pain in the lower jaw since 20 days. On extraoral examination there was very mild swelling in the left side angle of mandible. During intraoral examination there was buccal and lingual cortical expansion in the lower left jaw in relation to mandibular left canine, deciduous first and second molar (Fig. 1). Panoramic radiograph (Fig. 2) revealed multiple radiolucencies with scalloped border around unerupted right canine, left first premolar, right first and second premolars. Radiolucencies circumscribing mandibular second and third molar, notching the coronoid and condylar process on both side of the mandible are also evident. The mandibular left canine bud was missing. Multiple cystic spaces were clinically mimicking dentigerous cyst.

**Fig. 3:**
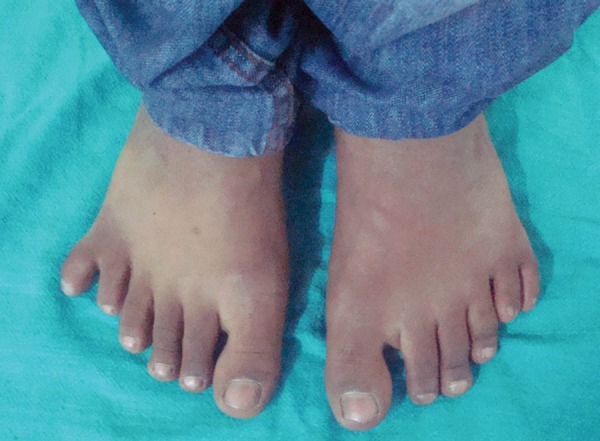
Polydactyly in both feet

**Fig. 4:**
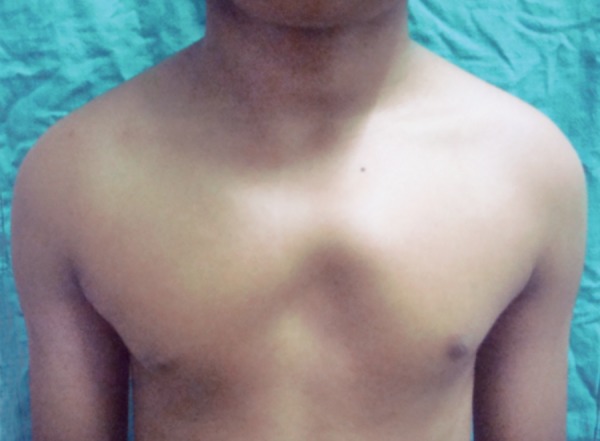
Pectus excavatum

**Fig. 5:**
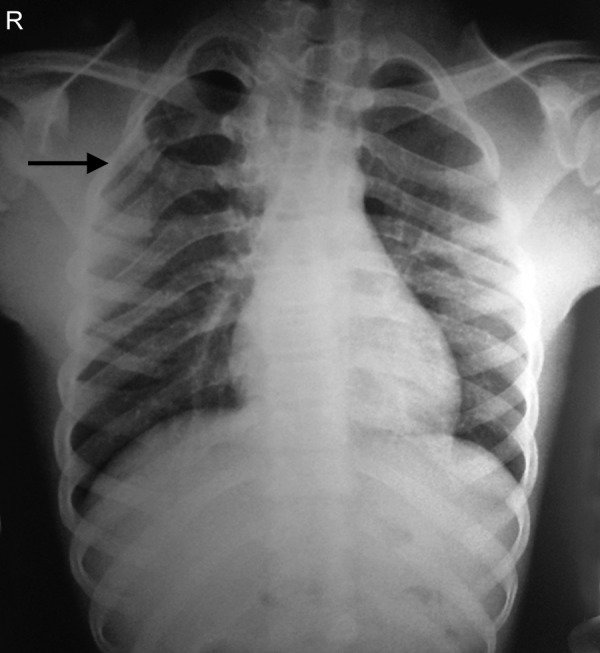
PA view chest X-ray showing vertebral rib deformity in the cervicodorsal region

**Fig. 6:**
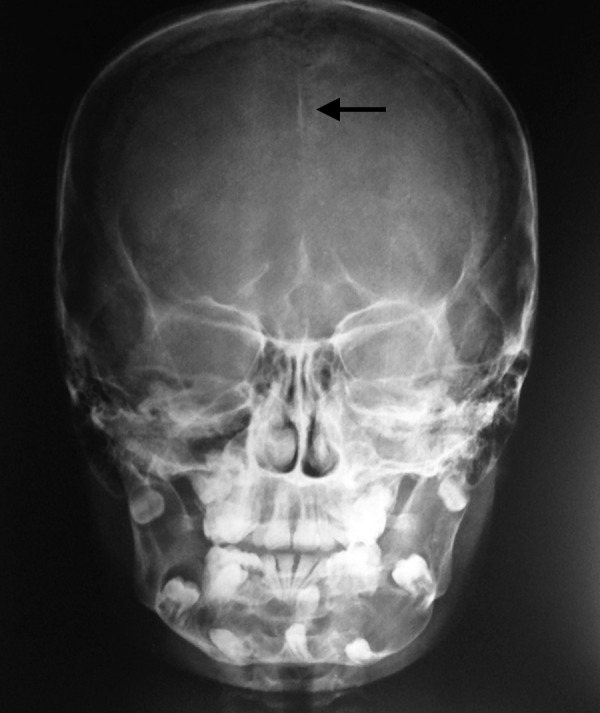
AP view of skull showing minor degree of falx cerebri calcification

On general examination we found polydactyly on both feet (Fig. 3), and pectus excavatum (Fig. 4). On taking the history the patient said he has undergone surgery for congenital cataract. The patient was advised to do posteroanterior (PA) view chest X-ray, PA view of skull and ultrasound of abdomen. PA view chest X-ray (Fig. 5) revealed vertebral rib deformity in the cervico dorsal region. PA view of skull showed minor degree of falx cerebri calcification (Fig. 6). There was no specific finding in the ultrasound of abdomen.

His family history was negative for any of the above mentioned findings. The pregnancy was uneventful and the child was born out of nonconsanguineous marriage.

**Fig. 7:**
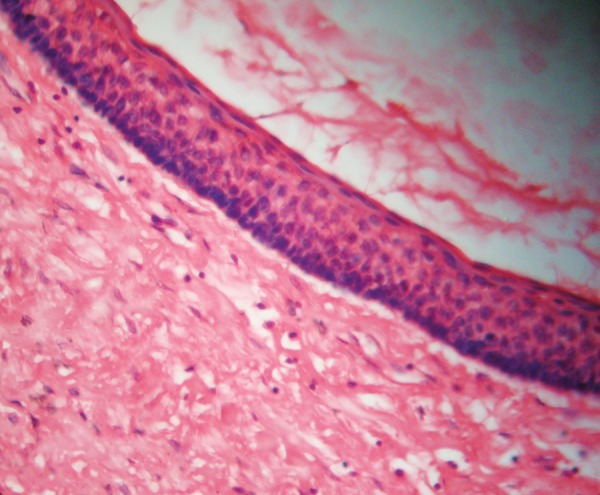
Histopathology 10× view

Marsupialization of the cysts were done under general anesthesia and sent for histopathology. Histopathological examination revealed (Fig. 7) parakeratinized stratified squamous epithelium of 6 to 10 cells thick. Tall columnar hyperchromatic basal cells were seen in a palisading manner. Corrugated keratin layer was seen superficially. A diagnosis of multiple odontogenic keratocyst was made.

## DISCUSSION

Gorlin-Goltz syndrome also known as nevoid basal cell carcinoma syndrome, described for the first time in 1894 by Jarisch and White.^11^ Global signs and symptoms associated with this syndrome were described in detail by Robert Gorlin and Robert Goltz in 1960,^12^ after which the condition became to be known as Gorlin-Goltz syndrome (GGS).

GGS is caused by germ line mutation of tumor suppressor gene (PTCH) on chromosome 9q22.3-q31; which was identified as being responsible for the developmental abnormalities and neoplasms.^13^ This gene is significant for embryonic structuring and cell cycle and thus its mutation comprises a key event for the development of the disease.

Evans et al^14^ first established major and minor criteria for diagnosis of this rare entity, later modified by Kimonis et al.^15^

### Major Criteria

- More than two basal cell carcinomas or one basal cell carcinoma at younger than 30 years of age or more than 10 basal cell nevi
- Any odontogenic keratocyst (proven on histology) or polyostotic bone cyst
- Three or more palmar or plantar pits (present in about 65% of patients)
- Ectopic calcification: lamellar or early at younger than 20 years of age
- Falx cerebri calcification
- Positive family history of nevoid basal cell carcinoma.
- Macrocephaly
- Congenital anomalies (cleft lip or palate, frontal bossing, coarse facies, and moderate or severe hypertelorism)
- Other skeletal anomalies (Sprengel deformity, marked pectus deformity, and marked syndactyly of the digits)
- Radiologic anomalies (e.g. bridging of the sella turcica, vertebral anomalies, flame-shaped lucencies of the hands and the feet.

Two major or one major and two minor criteria are obligatory for the diagnosis of Gorlin-Goltz syndrome.^15^This patient presented with multiple odontogenic keratocyst, Pectus excavatum, polydactyly, bifid rib, falx cerebri calcification and precocious cataract for which he had undergone surgery.

The most common and significant features of Gorlin-Goltz syndrome are summarized in Table 1.

The most consistent and representative sign is the presence of odontogenic keratocysts. Lo Muzio et al.^16^reported that OKC was the first symptom of Gorlin-Goltz syndrome in 78% of their cases. Among the various presentations, OKCs are often the first sign of Gorlin syndrome, frequently antedating the syndromic basal cell carcinomas, thereby allowing for earlier diagnosis.^16^ Based on the intrinsic growth potential of its epithelial coating, the most recent edition of WHO designates the OKC as a keratocystic odontogenic tumor,^17^ implying that the lesion is a benign neoplasm (KCOT). The molecular analysis of PTCH1 in Gorlin syndrome-associated tumors suggests that a ‘two-hit’ hypothesis is applicable to their pathogenesis.^18^These syndrome-associated OKCs presumably must have originated from precursor cells containing a hereditary ‘first hit’. Additional somatic mutation, loss of heterozygosity (LOH), or epigenetic silence of the other allele, may act as the ‘second hit’, which would make the PTCH1 protein functionally inactive.

**Table 1:** **Table 1:** Anomalies in Gorlin-Goltz syndrome

*Skeletal anomalies*		*Craniofacial anomalies*		*Neurological anomalies*		*Oropharyngeal anomalies*
Bifid ribs Splayed/fused ribs Cervical ribs Absent/rudimentary ribs (26%) Scoliosis Hemivertebrae Flame-shaped lucencies hand/feet Polydactyly Syndactyly Shortened 4th metacarpal		Frontal bossing (25%) Brachycephaly Macrocephaly (40%) ‘Coarse face’ (50%) Calcification of falces (37-79%) Tentorium cerebellum calcification Bridged sella turcica		Agenesis/disgenesis of corpus callosum Congenital hydrocephalus Mental retardation Medulloblastoma (3-5%) Meningioma (1% or less) Schizoid personality		High-arched palate or prominent palatineridges (40%) Odontogenic keratocysts (75%) Malocclusion(s) (maxillary hypoplasia and mandibular hyperplasia, cleftpalate) Dental ectopic position Impacted teeth and/or agenesis
*Skin anomalies*		*Sexual anomalies*		*Ophthalmic anomalies*		*Cardiac anomalies*
Basal cell carcinoma(50-97%)		Uterine and ovarian fibromas(15%)		Congenital amaurosis Exotropia		Cardiac fibroma (3%)
Palmar and/or plantar pits(90%)		Calcified ovarian cystsSupernumerary nippleHypogonadism andcryptorchidism		Hypertelorism (40%)PtosisInternal strabismus (15%)GlaucomaColobomaBlindness		

Like neoplasms in other cancer predisposition syndromes, OKCs in Gorlin syndrome patients are multiple and appear in a random pattern; similar isolated defects are also seen occasionally in the general population.^19^Syndrome-associated OKCs are to be found in both jaws with equal frequency, in contrast to sporadic OKCs, which involve especially the lower jaw.^20^ This report is contradicting the above statement.

Other findings include mandibular prognathism, malocclusion and dental agenesis. For the presence of possible satellite cysts solely surgical approach to the management of OKCs of NBCCS patients are unlikely to be successful. As a consequence adjunctive therapies, such as cryotherapy or Carnoy's solution are also indicated. Carnoy's solution causes protein coagulation and is safe and effective.^9^

Despite the name of the syndrome, multiple basal cell carcinomas (BCCs) occur only in 50% of the cases. They may vary in number from a few to 1000 and range in size from 1 to 30 mm in diameter. BCCs most often involve face and nonexposed areas, such as the back and chest. Patients with GGS are extremely sensitive to ionizing radiation and should be counseled to avoid sun exposure.^21^

Several ophthalmic manifestations of Gorlin-Goltz syndrome are reported in the general medical and dermatologic literature. These include periocular basal cell carcinoma, hypertelorism, strabismus, nystagmus, congenital cataract, uveal coloboma, and glaucoma.^9^ This patient presented with congenital cataract for which he has undergone surgery at the age of five.

Thoracic cage anomalies are common (43%) particularly bifid and fused ribs.^10^ Cage anomalies such as bifid, fused or splayed ribs may be present. Pectus excavatum was a manifestation in our patient.

A marked syndactyly of toes and polydactyly may rarely occur (3%).^10^ Rai^22^ has reported a case of Gorlin-Goltz syndrome where the patient presented with 6 fingers on right foot. Our patient has presented with 6 fingers on both feet. In light of the experience in this case, a finding of multiple cysts of the jaws constitutes a strong argument for a full investigation of the patient in order to rule out this syndrome. The oral surgeon as well as the radiologist would appear to have the best prospects for making an early diagnosis of this condition.

The importance of this syndrome lies in some of its serious prognoses. Ameloblastomatous^23^ and carcinomatous^24^ changes are known to have developed in the jaw cysts. The latter would emphasize the importance of complete surgical removal of the lining of jaw cysts and their subsequent histological examination. It is vital for patients to be referred to the department of genetics for counseling. Each child of an affected person has a one chance out of two of also inheriting the faulty gene and developing the signs of the conditions. Gene tracking and mutation analysis may be helpful for presymptomatic diagnosis.

## CONCLUSION

It is of relevant importance to examine the family of the patient diagnosed with Gorlin-Goltz syndrome to detect possible clinical manifestations and in that way arrive at an effective genetic advice. It is advisable to carry out all the necessary clinical and radiographic investigation in patients with multiple odontogenic keratocyst in the early stages of life, with the firm objective of making an early diagnosis of other possible associated malignant neoplasia which would be possible by considering the opinion of the different specialties such as dentists, maxillofacial surgeons, dermatologists, plastic surgeons, etc. and for this reason, basic experience of this syndrome is essential.
